# A204 COST-EFFECTIVENESS OF THERAPIES AFTER FAILURE OF CONVENTIONAL THERAPY FOR PATIENTS WITH MODERATE-TO-SEVERE ULCERATIVE COLITIS IN THE CANADIAN HEALTHCARE SYSTEM

**DOI:** 10.1093/jcag/gwac036.204

**Published:** 2023-03-07

**Authors:** C Hagerman, K I Kroeker, L Dieleman, F Peerani, D C Baumgart, K Wong, B Halloran

**Affiliations:** Division of Gastroenterology, University of Alberta, Edmonton, Canada

## Abstract

**Background:**

Ulcerative colitis (UC) is a chronic inflammatory disease of the colon which requires ongoing medical therapy. The therapeutic options for moderate-to-severe UC include biologics and small molecules, which are effective but come with a significant cost. As such, their exact positioning in the therapeutic algorithm remains unclear.

**Purpose:**

The aim of our study was to assess and compare the cost-effectiveness of infliximab, adalimumab, vedolizumab, golimumab, ustekinumab and tofacitinib for the management of moderate-to-severe UC from the perspective of the Canadian public healthcare system.

**Method:**

A Markov model was constructed to simulate the disease course of UC patients after initiating each available therapy. Drug costs were obtained from the Alberta Health Drug Benefit List and the remaining costs were determined from the CIHI Patient Cost Estimator. Transition probabilities were obtained from a review of the literature, and loss of response and complication rates were obtained from randomized controlled trials. Our main analysis used a time horizon of 5 years, and time horizons of 1- and 10-years were also assessed in our sensitivity analysis. Probabilistic sensitivity analysis was performed to characterize uncertainty related to all parameters.

**Result(s):**

Infliximab costs $26,611 per quality-adjusted life year (QALY) using a 5-year time horizon. Adalimumab costs $20,783 per QALY. Vedolizumab costs $40,553 per QALY. Golimumab costs $34,316 per QALY. Ustekinumab costs $26,366 per QALY. Lastly, tofacitinib costs $25,572 per QALY. At a willingness-to-pay threshold of $50,000 per QALY, sensitivity analysis revealed that infliximab, adalimumab, vedolizumab, golimumab, ustekinumab and tofacitinib had a 36%, 12%, 1%, 1%, 44% and 6% probability of being cost-effective, respectively.

**Conclusion(s):**

Our economic model concluded that adalimumab is the most cost-effective first-line therapy for UC patients who have failed conventional therapy.

**Please acknowledge all funding agencies by checking the applicable boxes below:**

None

**Disclosure of Interest:**

None Declared

